# MHC mediates social odor via microbiota—it cannot work: a comment on Schubert et al.

**DOI:** 10.1093/beheco/arab017

**Published:** 2021-05-18

**Authors:** Manfred Milinski

**Affiliations:** Max-Planck-Institute of Evolutionary Biology, August-Thienemann-Str. 2, Plön, Germany

Schubert et al. (2021) review the evidence from 577 publications about how the Major Histocompatibility Complex (MHC), might mediate social odor via the microbiota community to “stimulate advances in our knowledge of this key pathway for social communication.” The idea is that, as part of the immune system of all vertebrates, polymorphic MHC molecules control microorganisms present in the microbiome, which produce odor that may serve as a social signal. MHC, microbe, odor signal is the sequence of steps leading eventually from MHC to social signal, for example, for MHC-dependent mate choice. However, none of the 577 studies showed the odor to be a social signal.

MHC-dependent olfactory signaling helps females choose a mate that offers MHC alleles optimally complementing her own alleles to maximize resistance of her offspring. Male body odor signals the MHC immune alleles he can offer. Females compare his signal to their own MHC alleles and mate, if the male’s alleles are complementary. If microorganisms provide the odor signal, the few relevant signals should stand out from numerous irrelevant odors from the whole microbiome. The female needs to tell exactly those odors that signal the possession of the male’s few MHC alleles that affect his immune response against parasites and microbes. If she can tell, she has to know exactly which MHC allele each odor depicts. She needs this knowledge for all odors of the microbiome that can signal MHC alleles. How can a female acquire this knowledge? It is extremely unlikely that she can. The next problem: the immunogens that a female needs to uncover do their job and eliminate exactly those microorganisms as intruders; the “wanted” microorganisms no longer signal, they have been killed. Bolnick et al. (2014) showed a negative correlation between MHC diversity and microbial diversity supporting the hypothesis that a diverse MHC genotype causes elimination of more microbiota species. The idea could be rescued by assuming that the female knows the odors of all microorganisms of a potential mate and deduces those that are missing. It would be the virtual signals of the gaps she needs to take into account for mate choice, an obviously unsolvable problem.

As the authors further suggest, the MHC might tolerate microorganisms involved in MHC signaling. No doubt, keeping one’s symbionts increases fitness. The interaction between the microbiota and the host are influenced by host genetics, cooperation and competition between pathogenic and commensal microbes and multiple environmental variables, including diet, circadian factors and climate (Honda and Littman 2016). Mucosal IgA secreted across the epithelium binds to microbes, various components of the diet and to antigens. This averts potentially harmful stimulation of the immune system and serves to regulate the composition of the microbiota increasing their diversity (Honda and Littman 2016). Thus, the highly variable diversity of tolerated microbiota precludes toleration of only, and exactly, those symbionts that might signal a male’s MHC alleles, in addition to the problem of uncovering the MHC alleles’ identity from the symbionts’ odor.

For my third criticism, I refer to an established mechanism of MHC signaling. An optimal individual number of MHC alleles (molecules) exists, which maximizes resistance to parasites, shown experimentally in sticklebacks (Wegner et al. 2003), and later for other vertebrates. To provide the offspring with this optimal MHC through mate choice, there must be odor cues that signal the male’s MHC alleles. The female determines from those cues whether they optimally complement her own MHC alleles. When detected she prefers such a male almost perfectly (Milinski et al. 2005). Proteins of parasites and microbes are degraded to peptides. The MHC odor cues have been identified as the peptides that are bound to MHC molecules and presented to T-cells: Because of a key-lock relationship between the binding specificity of the MHC molecule and the anchor residues of the peptide (Boehm and Zufall 2006), the peptide depicts the identity of the MHC molecule, deciphered by specialized odor receptor neurons in the vomeronasal organ (Leinders-Zufall et al. 2004) and the main olfactory epithelium (Spehr et al. 2006). An experimental proof of the specific signal function of peptides was provided by using synthesized nature-identical peptides that altered, when added to the male’s signal, predictively the mate choice decision of the female (Milinski et al. 2005). Probably all jawed vertebrates signal MHC alleles with peptides because the vertebrate immune system is highly conserved.

Using a signaling mechanism, such as assumed social odor from microorganisms, that transmits the same information as an existing one, does not increase the sender’s fitness. It would thus not evolve. Surviving microbiota produce odor as a side effect of their existence, with no relevance for MHC signaling.
